# Genetic mapping of leaf rust (*Puccinia triticina* Eriks) resistance genes in six Canadian spring wheat cultivars

**DOI:** 10.3389/fpls.2023.1130768

**Published:** 2023-03-20

**Authors:** Firdissa E. Bokore, Richard D. Cuthbert, Ron E. Knox, Colin W. Hiebert, Curtis J. Pozniak, Samia Berraies, Yuefeng Ruan, Brad Meyer, Pierre Hucl, Brent D. McCallum

**Affiliations:** ^1^ Swift Current Research and Development Centre, Agriculture and Agri-Food Canada, Swift Current, SK, Canada; ^2^ Morden Research and Development Centre, Agriculture and Agri-Food Canada, Morden, MB, Canada; ^3^ Department of Plant Sciences, University of Saskatchewan, Saskatoon, SK, Canada

**Keywords:** *Triticum aestivum*, *Puccinia triticina*, seedling resistance, QTL, SNP markers

## Abstract

The Canada Western Red Spring wheat (*Triticum aestivum* L.) cultivars AAC Concord, AAC Prevail, CDC Hughes, Lillian, Glenlea, and elite line BW961 express a spectrum of resistance to leaf rust caused by *Puccinia triticina* Eriks. This study aimed to identify and map the leaf rust resistance of the cultivars using three doubled haploid populations, AAC Prevail/BW961 (PB), CDC Hughes/AAC Concord (HC), and Lillian/Glenlea (LG). The populations were evaluated for seedling resistance in the greenhouse and adult plant disease response in the field at Morden, MB for 3 years and genotyped with the 90K wheat Infinium iSelect SNP array. Genetic maps were constructed to perform QTL analysis on the seedling and field leaf rust data. A total of three field leaf rust resistance QTL segregated in the PB population, five in the HC, and six in the LG population. In the PB population, BW961 contributed two QTL on chromosomes 2DS and 7DS, and AAC Prevail contributed a QTL on 4AL consistent across trials. Of the five QTL in HC, AAC Concord contributed two QTL on 4AL and 7AL consistent across trials and a QTL on 3DL.1 that provided seedling resistance only. CDC Hughes contributed two QTL on 1DS and 3DL.2. Lillian contributed four QTL significant in at least two of the three trials on 2BS, 4AL, 5AL, and 7AL, and Glenlea two QTL on 4BL and 7BL. The 1DS QTL from CDC Hughes, the 2DS from BW961, the 4AL from the AAC Prevail, AAC Concord, and Lillian, and the 7AL from AAC Concord and Lillian conferred seedling leaf rust resistance. The QTL on 4AL corresponded with *Lr30* and was the same across cultivars AAC Prevail, AAC Concord, and Lillian, whereas the 7AL corresponding with *LrCen* was coincident between AAC Concord and Lillian. The 7DS and 2DS QTL in BW961 corresponded with *Lr34* and *Lr2a*, respectively, and the 1DS QTL in CDC Hughes with *Lr21*. The QTL identified on 5AL could represent a novel gene. The results of this study will widen our knowledge of leaf rust resistance genes in Canadian wheat and their utilization in resistance breeding.

## Introduction

Wheat (*Triticum aestivum* L.) leaf rust, caused by the obligate biotrophic pathogen *Puccinia triticina* Eriks. (*Pt*), is one of the most destructive and prevalent diseases of wheat worldwide (Kolmer, 2005). Wheat leaf rust disease has been an annual problem since the early days of wheat cultivation in Canada and other countries (Kolmer, 2005; McCallum et al., 2007; McCallum et al., 2016a). The use of fungicides and breeding to combine genetic resistance is the most common method to prevent and control wheat leaf rust (Hafeez et al., 2021). Genetic resistance is the most preferred and effective method to combat *Pt* and reduce yield loss. In Canada, for the last several years, leaf rust has been effectively controlled by growing highly resistant cultivars. Wheat farmers could continue to economically benefit from growing disease-resistant cultivars with an increase in farm income through yield protection and a reduction in farm expenditure because of the absence or reduced application of fungicides. However, the efficacy of some resistance genes, such as *Lr21*, has decreased over time, and other genes could be overcome by evolving races resulting from the mutation and selection of virulent pathogen genotypes, suggesting the need for the continuous search and deployment of new and effective genes.

For the past many decades, several leaf rust-resistant wheat cultivars have been developed and deployed in different countries around the world. The identification of *Lr* genes effective in the adapted wheat germplasm and wild relatives to introgress into new cultivars has been a major breeding objective of many wheat programs. Two classes of *Lr* genes have been known to condition the resistance to leaf rust in wheat—(1) the R genes also referred to as “major gene resistance,” “gene-for-gene resistance,” “race specific resistance,” “qualitative resistance,” and “seedling or all-stage resistance” and (2) “adult plant resistance,” “quantitative resistance,” “slow rusting,” or “durable resistance” genes (Kolmer, 1997; Ellis et al., 2014; Singh et al., 2016).

While R genes are pathogen race-specific and express throughout the stages of the plant growth, the expression of the adult plant resistance (APR) genes is usually confined to adult plants. The R genes confer complete resistance compared with some adult plant resistance genes, which confer partial and more durable protection against the disease, while other adult plant resistance genes are also race-specific. In general, cultivars with only adult plant resistance are susceptible to infections as seedlings but become more resistant as the plant grows. Leaf rust resistance genes designated *Lr1* to *Lr81* and several quantitative trait loci (QTL) associated with the resistance have been described (Pinto da Silva et al., 2018; McIntosh et al., 2020; Kumar et al., 2021; Kumar et al., 2022; Xu et al., 2022). Pinto da Silva et al. (2018) reported 249 leaf rust resistance QTL identified in 70 biparental mapping populations and 79 different lines, 35 meta-QTL, and about 200 marker-trait associations (MTAs) identified on the 21 wheat chromosomes. However, most designated genes are no longer used in breeding as they are not effective against the recent *Pt* races.

Breeding durable resistance in wheat cultivars depends on the continuous introgression of new genes into adapted cultivars (Kolmer, 1997; Campbell et al., 2012). Pyramiding of multiple genes is an effective approach utilized in resistance breeding. For example, inheritance studies conducted using CIMMYT wheat germplasm by Singh et al. (2000) indicated that combinations of three to five small- to intermediate-effect genes could result in a high level of resistance. Several other studies also affirm stacking genes could enhance the level of resistance, subsequently increasing the longevity of cultivars in the field (Kolmer, 1997; Singh et al., 2016; Mundt, 2018; Rimbaud et al., 2018; Hafeez et al., 2021; Bokore et al., 2022). Additionally, growing cultivars strategically pyramided with resistance genes could help in restricting the chance of evolving virulent races.

Molecular characterization of wheat germplasm is useful to identify new genes, track previously reported genes, develop markers suitable for marker-assisted breeding, and identify parental lines that could be used to develop new cultivars. The objective of this study was to identify and map leaf rust resistance in the Canadian spring wheat cultivars AAC Prevail, AAC Concord, CDC Hughes, Glenlea, Lillian, and elite line BW961 using three doubled haploid populations derived from these lines.

## Materials and methods

### Plant materials

**Figure 1:**
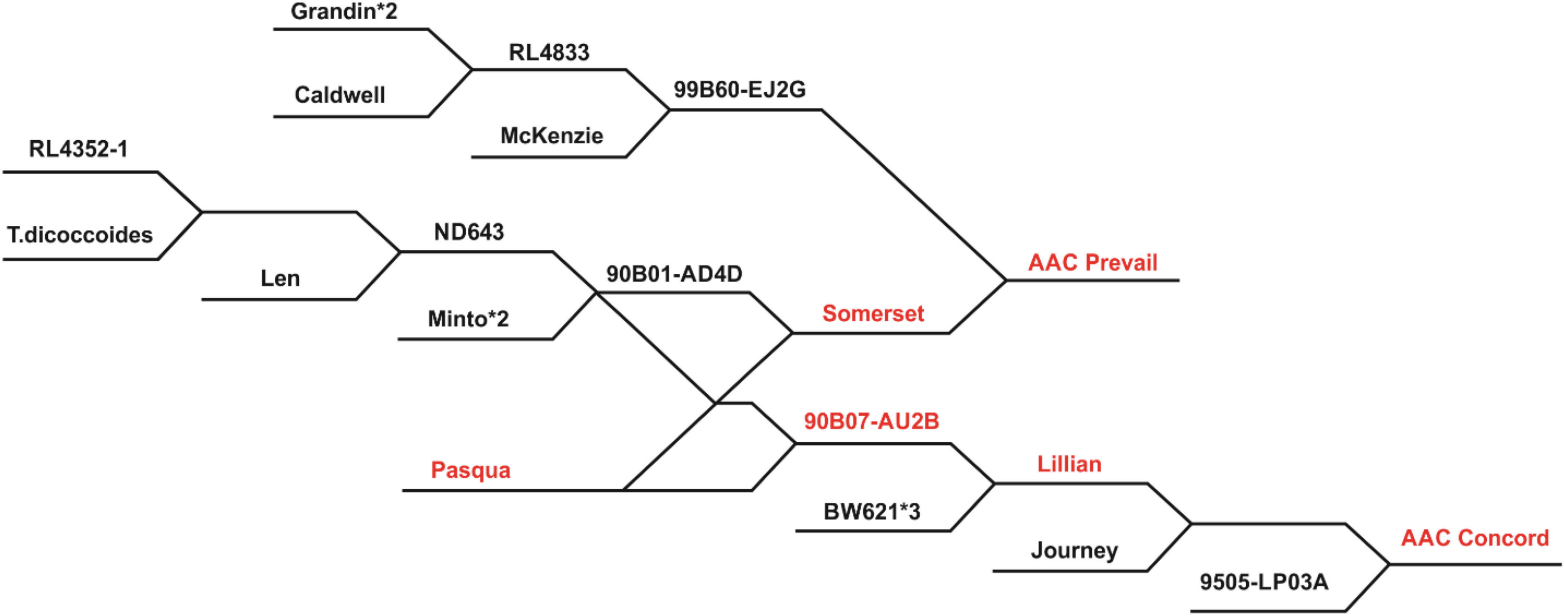
A dendrogram displaying Pasqua descendants Lillian, AAC Concord, and AAC Prevail. The asterisk sign “*” followed by numbers indicates how many times a recurrent parent was crossed with a line. RL4352-1 is a rust-resistant selection from cv. Columbus (Basnet et al., 2015). Pasqua descendants (red font).

Three doubled haploid (DH) populations developed from the CWRS wheat crosses AAC Prevail/BW961 (PB), CDC Hughes/AAC Concord (HC), and Lillian/Glenlea (LG) using the wheat–maize–pollen method (Humphreys and Knox, 2015) at AAFC-Swift Current, SK, were studied. **Table 1** describes population size, location, and year of leaf rust evaluation and a number of lines genotyped with the 90K wheat Infinium iSelect single nucleotide polymorphism (SNP) array. Derived from the cross 99B60-EJ2G/Somerset, AAC Prevail (**Figure 1**) was granted breeders’ right in 2017, and it was rated resistant to leaf rust at the time of its registration (Kumar et al., 2017). Lillian, a line derived from a cross BW621*3/90B07-AU2B (**Figure 1**), is a solid stem cultivar registered in 2003 with a resistant response to prevalent races of leaf rust at the time of its release (DePauw et al., 2005). AAC Concord, released in 2017 and selected from the cross Lillian/Journey//9505-LP03A (**Figure 1**), was resistant to leaf rust when it was registered (https://inspection.canada.ca/english/plaveg/pbrpov/cropreport/whe/app00009998e.shtml). Licensed in 1972, Glenlea was also resistant to all prevalent races of leaf rust at the time of its release (Evans et al., 1972). With a moderate resistance to leaf rust during its registration, CDC Hughes, provisionally protected in 2016 and granted rights in 2019, was developed from the cross Unity/BW864 (https://inspection.canada.ca/english/plaveg/pbrpov/cropreport/whe/app00010339e.shtml). Having moderate leaf rust resistance, BW961 is a breeding line selected from Alsen/Waskada, but it was not registered as a cultivar (https://inspection.canada.ca/english/plaveg/pbrpov/cropreport/whe/app00009618e.shtml).

**Table 1:** Description of the doubled haploid wheat populations AAC Prevail/BW961, CDC Hughes/AAC Concord, and Lillian/Glenlea used in the leaf rust resistance study, the number of lines genotyped and phenotyped near Morden (MD), Manitoba, and the year-of-field evaluation.

Cross	Population size		SNP genotype	Location × year
	Phenotyped	Genotyped		
AAC Prevail/BW961	227	227	5K	MD2019, 2020, 2021
CDC Hughes/AAC Concord	204	188	90K	MD2019, 2020, 2021
Lillian/Glenlea	191	190	90K	MD2019, 2021, 2022

### Leaf rust evaluation

#### Seedling plant infection

The seedling infection type response analysis on the parents and progenies of the PB, HC, and LG populations was conducted in the greenhouse at Morden Research and Development Centre, AAFC, MB, using *Pt* isolates 12-3 MBDS, 128-1 MBRJ, 74-2 MGBJ, 06-1-1 TDBG, 77-2 TJBJ, and 21-84-1TCTS. The isolates have unique numerical identifiers, and the letter codes are according to the North American system of nomenclature described by Long and Kolmer (1989). The seedlings were inoculated by urediniospores of single purified *Pt* isolates at the two-leaf growth stage, as previously described by McCallum et al. (2020). Infected plants were rated to determine the infection type (IT) 12 to 14 days post-inoculation (McCallum et al., 2020). Wheat lines that produced infection types “;” (hypersensitive flecks), “1” (small uredinia with necrosis), “2” (small- to medium-sized uredinia with chlorosis), and “*X*” or mesothetic (a range of reaction types from “;” to “3−”) were considered resistant, and those that produced infection types “3” (medium-sized uredinia without chlorosis or necrosis) and “4” (large uredinia without chlorosis or necrosis) were considered susceptible against the *Pt* races evaluated (Stakman et al., 1962). Pustules that were slightly larger than typical for the infection were designated with a “+,” and those slightly smaller were designated with “−,” whereas those much smaller than the typical infection type were designated with “=.” For QTL analysis, the seedling infection types were converted in a similar manner to a 1 to 9 scale as previously described (Zhang et al., 2011; Zhang et al., 2014). The ITs were converted as follows: “0” = 0, “1−”=1, “1” = 2, “1+”=3, “2−”=4, “2” = 5, “2+”=6, “*X*”=7, “3−”=8, “3” = 9, and “3+”=10 and “4” = 10.

#### Adult plant infection

Trials to evaluate the adult plant leaf rust responses of the populations were conducted in the field rust nurseries near Morden, MB. The populations of PB and HC were evaluated for 3 years from 2019 to 2021, and the LG population in 2019, 2021, and 2022. The populations, parents, and checks were planted in single 1-m rows in a randomized complete block design with two replications. To ensure uniform infestation, susceptible spreader rows were planted around plots, and the spreader rows were inoculated with a mixture of Canadian leaf rust races collected during the previous growing season (McCallum et al., 2016b; Bokore et al., 2020; McCallum et al., 2021). The urediniospores of multi-race mixtures were suspended in light mineral oil (Soltrol, Chevron Phillips Chemical Company) and sprayed on the leaves of the spreader rows at early tillering. This enables the urediniospores to develop on the spreader rows and be windblown to the test lines, providing a uniform rust infection across the field.

Leaf rust severity was rated as the proportion (%) of the leaf area infected using a modified Cobb Scale (Peterson et al., 1948) and infection response as resistant (*R*), resistant to moderately resistant (RMR), moderately resistant (MR), mesothetic (*X*), moderately resistant to moderately susceptible (MRMS), moderately susceptible (MS), moderately susceptible to susceptible (MSS), and susceptible (*S*). The infection response was converted to numerical values for data analysis as *R* = 1, RMR = 2, MR = 3, *X* = 4, MRMS = 5, MS = 6, MSS = 7, and *S* = 8. Pearson correlation analysis on the disease severity of adult plants was performed using SAS 9.4 software.

### Molecular analysis

The DNA of the DH lines and parents of the PB, HC, and LG populations was extracted from young leaves using the DNeasy 96 Plant Kit (QIAGEN Science, MD, USA). The PB population was genotyped with 5,151 SNP markers chosen for wheat breeding from the 90K Infinium ISelect marker panel, as previously described by Bokore et al. (2021). The HC and LG populations and parental lines were genotyped using the 90K Infinium iSelect SNP bead array (Illumina Inc., San Diego, CA, USA). The SNP raw data of each population were processed in GenomeStudio v2.0 software (Illumina). The linkage maps of the populations were built using the regression model in JoinMap 5 software, Kyazma, Wageningen, The Netherlands (Van Ooijen, 2018). The QTL analysis was performed on the seedling and adult plant leaf rust phenotypic data using the MapQTL 6 software, Kyazma, Wageningen, The Netherlands (Van Ooijen, 2009). The analysis of both seedling and adult plant phenotypic data along with the chromosomal locations of the QTL enabled the assignment of most QTL to known leaf rust genes. The multiple QTL mapping (MQM) methods were adopted to confirm QTL regions first detected by the simple interval mapping approach. Cofactor markers used in the MQM analysis were selected by automatic cofactor selection or manually by adjusting the selection of markers.

## Results

### Parental line seedling plant response

The PB population parent AAC Prevail evaluated with *Pt* races 12-3 MBDS, 128-1 MBRJ, 74-2 MGBJ, 06-1-1 TDBG, and 77-2 TJBJ displayed resistant IT of “;1=“ against all the races. The second PB population parent, BW961, displayed resistant IT of “0” against races 12-3 MBDS, 128-1 MBRJ, and 74-2 MGBJ and intermediate IT of 2 to 3 against 06-1-1 TDBG and 77-2 TJBJ. The first HC parent, CDC Hughes, was rated with an IT of “;1=“ for the races 12-3 MBDS, 128-1 MBRJ, 06-1-1 TDBG, and 77-2 TJBJ and an IT of “;” against 74-2 MGBJ. The second HC population parent, AAC Concord, was rated with an IT of “0” for the races 12-3 MBDS, 128-1 MBRJ, 74-2 MGBJ, an IT of “;” for 06-1-1 TDBG, and an IT of “;1” for 77-2 TJBJ. The LG parent Lillian was rated with an IT of “;1” against the races 12-3 MBDS, 74-2 MGBJ, and 77-2 TJBJ, with an IT of a mixture of “2+” and “*X*” against 128-1 MBRJ, an IT of “;” with the race 06-1-1 TDBG, and an IT of “2” with 21-84-1 TCTS. The other LG population parent, Glenlea, had intermediate IT of “2” to “3” for race 128-1 MBRJ, susceptible with an IT of “3” for 12-3 MBDS and 74-2 MGBJ, had an IT of “3+” for 06-1-1 TDBG and 21-84-1 TCTS, and was highly susceptible with an IT of “4” for 74-2 MGBJ.

### Field adult plant response

**Figure 2:**
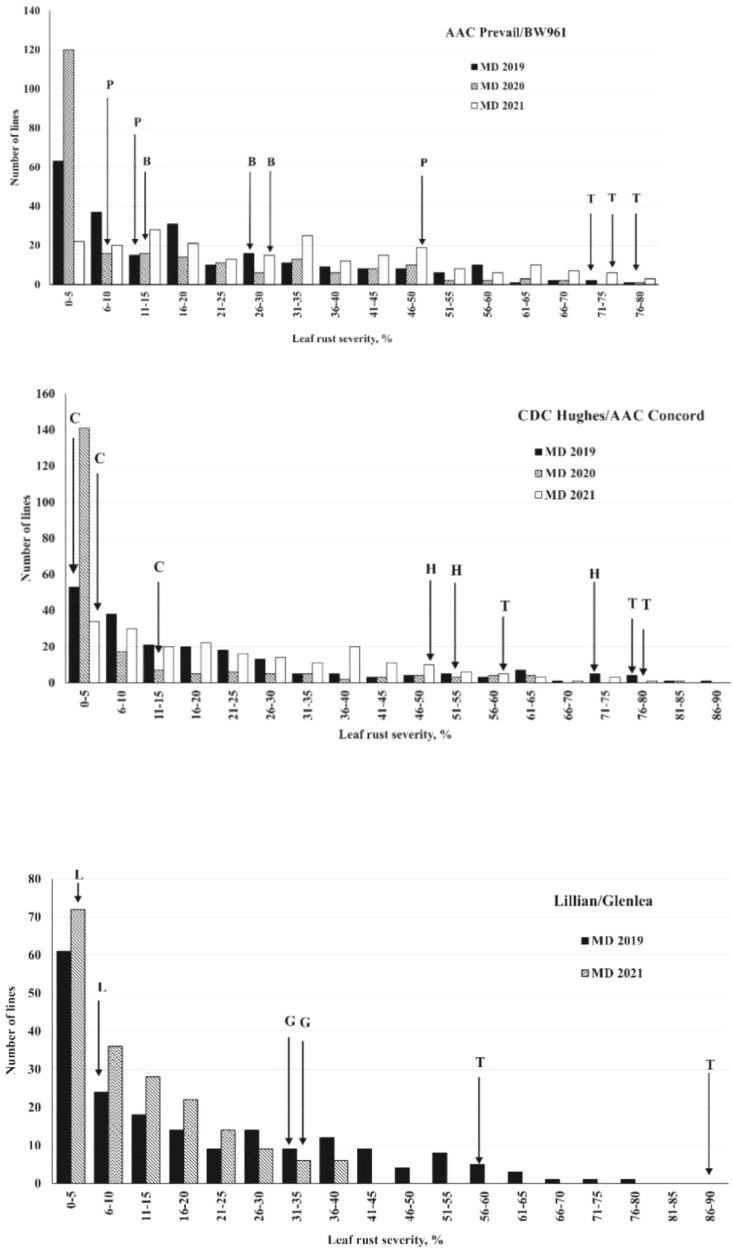
Frequency distribution of leaf rust severity for lines derived from spring wheat crosses AAC Prevail/BW961, CDC Hughes/AAC Concord, and Lillian/Glenlea. Cultivar or line name abbreviations: L, Lillian; G, Glenlea; P, AAC Prevail; B, BW961; C, AAC Concord; H, CDC Hughes; T, Thatcher.

A wide range of percent disease severity and infection response (*R* to *S*) was observed among the DH lines of the populations (**Supplemental Table S1**). The frequency distribution of the leaf rust severity among lines of the populations is presented in **Figure 2**. Across all the trials and environments, the susceptible check Thatcher ranged in leaf rust severity from 58.8% in 2021, with dry and hot weather too, to as high as 90.0% severity in 2019. The first PB population parent, AAC Prevail, showed 13.3% leaf rust severity in 2019, 7.5% in 2020, and 46.9% in 2021 with MR to MRMS infection response compared with the second parent BW961, which displayed 25.8% leaf rust severity in 2019, 13.1% in 2020, and 28.1% in 2021 with MRMS to MS infection response. The HC parent, AAC Concord, showed 0.8% to 13.8% disease severity with RMR to MR response compared with the other parent, CDC Hughes, which had high severity ranging from 47.5% to 72.5% and MRMS to *S* response across environments. The LG parent, Lillian, displayed high resistance with 2% to 13.8% disease severity and R-MRMS infection response, whereas the other parent, Glenlea, showed 31.3% to 38.8% disease severity with MRMS to *S* infection response across environments. Pearson correlation coefficients (**Table 2**) between the different environments for the leaf rust severity of the three populations ranged from intermediate to high with a range of 0.67 to 0.82 for the PB population, 0.62 to 0.84 for the HC, and 0.51 to 0.75 for the LG population.

**Table 2:** Pearson correlation coefficients (*r*) between leaf rust severity for different environments in three populations.

**AAC Prevail/BW961**	**MD2019** ^a^	**MD2020**
MD2020	0.82°	–
MD2021	0.67	0.67
**CDC Hughes/AAC Concord**	MD2019	MD2020
MD2020	0.84	–
MD2021	0.65	0.62
**Lillian/Glenlea**	MD2019	MD2021
MD2021	0.60	–
MD2022	0.75	0.51

a Test environments designated as MD for Morden, MB followed by a year-of-field evaluation. For example, MD2019 designates the experiment conducted at Morden, MB in 2019.

°Significant at p < 0.0001.

### Genetic linkage map

The SNP genotype and genetic map of the three populations are given in **Supplemental Table S2**. The genetic map of the PB population consisted of 5,151 breeding SNP array markers previously reported by Bokore et al. (2021). From the 5,151 breeding SNP array used in genotyping the population, 963 SNP markers showed polymorphism between parents and were used to construct a genetic map of the population. The 963 markers covered a total length of 2,898.8 cM with 32 linkage groups across all the hexaploid wheat genomes except chromosome 4D. The 90K SNP genotyping of the HC population produced a total of 9,881 valuable SNP markers used to map the population. Out of the 9,881 markers, 9,648 polymorphic markers were mapped to 27 linkage groups, and 4,080 (42.3%) of the markers were mapped to chromosome group A, 4,714 (48.9%) to chromosome group B, and 854 (8.9%) to group D. The total length of all the linkage groups was 2,920.1 cM with a density of 0.5 markers/cM. For the LG population, 7,293 valuable markers were mapped to 39 linkage groups covering 4,994.7 cM of the wheat genome, with 2,839 (38.9%) markers being assigned to chromosome group A, 3,617 (49.6%) markers to chromosome group B, and 837 (11.3%) markers assigned to group D.

### QTL detected using seedling plant response data

The seedling data revealed two significant QTL that were similarly effective on adult plants in the field in the PB population (**Tables 3**, **4**). The resistance allele for the first QTL was inherited from BW961 on chromosome 2DS, and it was detected with *Pt* races 12-3 MBDS, 128-1 MBRJ, and 74-2 MGBJ. The second QTL was inherited from AAC Prevail and detected on chromosome 4AL with races 74-2 MGBJ, 06-1-1 TDBG, and 77-2 TJBJ. The QTL on 4AL explained phenotypic variation in the seedling reaction ranging from 10.6% to 18.5% across the *Pt* races, and the 2DS QTL explained 31.8% to 44.9% of the variation across the *Pt* races.

**Table 3:** Leaf rust resistance QTL identified in the AAC Prevail (P)/BW961 (B), CDC Hughes (H)/AC Concord (C), and Lillian (L)/Glenlea (G) doubled haploid populations evaluated for seedling plant leaf rust infection response to isolates 12-3 MBDS, 128-1 MBRJ, 74-2 MGBJ, 77-2 TJBJ, 06-1-1 TDBG, 21-21-1 TNBJ, and adult plant disease response in nurseries located in Morden (MD), Manitoba in 2019, 2020, and 2021.

QTL/Gene	Trait name^a^	Environnent /*Pt* isolate	Peak markers	Peak position, cM	Start position^b^	LOD	PVE, %	Source of resistance allele^c^
***PB population***	*** ***	*** ***	* *					
*QLr.Spa-2D*	SEV	MD19	*RAC875_c65419_229*	95.8	63,714,903	2.1	4.2	B
	SEV	MD20	*RAC875_c65419_229*	95.8		2.3	4.5	B
	SEV	MD21	*RAC875_c65419_229*	95.8		4.7	9.1	B
	IR	MD21	*RAC875_c65419_229*	95.8		2.3	4.5	B
	IT	12-3 MBDS	*Kukri_c16477_181*	95.8	64,456,345	19.9	35.9	B
	IT	128-1 MBRJ	*Kukri_c16477_181*	95.8		28	44.9	B
	IT	74-2 MGBJ	*wsnp_Ku_c3107_5818628*	96.2		17.6	31.8	B
*QLr.Spa-4A*	SEV	MD19	*Tdurum_contig32577_286*	63.8	569,238,984	9.0	16.6	P
	IR	MD19	*Tdurum_contig32577_286*	63.8		9.5	17.6	P
	SEV	MD20	*Tdurum_contig32577_286*	63.8		6.3	12.0	P
	IR	MD20	*Tdurum_contig32577_286*	63.8		2.1	5.8	P
	SEV	MD21	*Tdurum_contig32577_286*	63.8		6.5	12.3	P
	IR	MD21	*Tdurum_contig32577_286*	63.8		8.3	15.4	P
	IT	74-2 MGBJ	*Tdurum_contig32577_286*	63.8		4.3	6.0	P
	IT	06-1-1 TDBG	*Tdurum_contig32577_286*	63.8		5.5	10.6	P
	IT	77-2 TJBJ	*Tdurum_contig32577_286*	63.8		3.4	8.3	P
*QLr.Spa-7D*	SEV	MD19	*BobWhite_c40479_283*	0.0	59,988,343	5.7	11.0	B
	IR	MD19	*BobWhite_c40479_283*	0.0		5.4	10.3	B
	SEV	MD20	*BobWhite_c40479_283*	0.0		6.9	13.1	B
	SEV	MD21	*BobWhite_c40479_283*	0.0		8.9	16.5	B
	IR	MD21	*BobWhite_c40479_283*	0.0		6.7	12.6	B
***HC population***	*** ***	*** ***	*** ***					
*QLr.spa-1D*	IT	12-3 MBDS	*RAC875_c48669_292*	107.5	172,996	4.8	11.5	H
	IT	128-1 MBRJ	*RAC875_c48669_292*	107.5		8.9	20.9	H
	IT	74-2 MGBJ	*BS00093336_51*	107.5	172,745	4.5	11	H
	IT	06-1-1 TDBG	*RAC875_c48669_292*	107.5		10.3	24.5	H
	IT	77-2 TJBJ	*RAC875_c48669_292*	107.5		18.1	38.8	H
	SEV	MD20	*RAC875_c48669_292*	107.5		2.2	5.2	H
	IR	MD20	*RAC875_c48669_292*	107.5		2.2	5.1	H
	SEV	MD21	*RAC875_c48669_292*	107.5		6.3	14.4	H
	IR	MD21	*RAC875_c48669_292*	107.5		8.5	18.7	H
*QLr.spa-3D.1*	IT	128-1 MBRJ	*Excalibur_c212_1553*	23.4	610,460,049	20.1	32.3	C
	IT	74-2 MGBJ	*Excalibur_c20595_614*	24.0	730,566,652	4.7	11.6	C
	IT	12-3 MBDS	*Kukri_c23354_183*	24.0		6.0	14.2	C
*QLr.spa-3D.2*	SEV	MD19	*Kukri_c31733_290*	13.0	301,139,033	4.4	10.1	H
	IR	MD19	*Kukri_c31733_290*	13.0		3.0	7.1	H
	SEV	MD20	*Kukri_c31733_290*	13.0		2.5	5.9	H
	IR	MD20	*Kukri_c31733_290*	13.0		2.9	6.8	H
	SEV	MD21	*Kukri_c31733_290*	13.0		2.3	5.3	H
*QLr.spa-4A*	IT	12-3 MBDS	*wsnp_Ex_c2266_4247520*	52.2	522,005,468	5.3	12.7	C
	IT	06-1-1 TDBG	*Kukri_rep_c102142_525*	50.2		7.9	19.4	C
	IT	77-2 TJBJ	*Ex_c70424_465*	51.6	476,083,357	25.3	30.4	C
	IT	21-21-1 TNBJ	*wsnp_Ex_c2266_4247520*	52.2		46.2	68.9	C
	SEV	MD19	*Kukri_rep_c102142_525*	50.6		10.9	23.5	C
	IR	MD19	*Kukri_rep_c102142_525*	50.6		7.8	17.3	C
	SEV	MD20	*Kukri_rep_c102142_525*	50.6		6.7	15.1	C
	IR	MD20	*Kukri_rep_c102142_525*	50.6		9.4	20.5	C
*QLr.spa-7A*	IT	06-1-1 TDBG	*RAC875_c90330_82*	211.1	727,373,599	5.2	13.3	C
	SEV	MD19	*BS00068033_51*	211.1	727,564,908	4.1	9.5	C
	IR	MD19	*BS00068033_51*	211.1		1.2	2.8	C
	SEV	MD20	*BS00068033_51*	211.1		3.5	8.3	C
	IR	MD20	*BS00068033_51*	211.1		2.4	5.8	C
	SEV	MD21	*BS00068575_51*	211.1		2.1	4.9	C
* *	IR	MD21	*BS00068033_51*	211.1		1.6	3.9	C
***LG population* **	*** ***	*** ***	** **					
*QLr.spa-2B*	SEV	MD19	*IAAV1101/BS00022966_51*	143.8	103,454,166	6.2	14.0	L
	IR	MD19	*IAAV1101/BS00022966_51*	143.8		6.1	13.8	L
	SEV	MD21	*IAAV1101/BS00022966_51*	143.8	103,454,166	4.3	9.8	L
	IR	MD21	*IAAV1101/BS00022966_51*	143.8		3.1	7.2	L
	SEV	MD22	*IAAV1101/BS00022966_51*	143.8		7.4	16.3	L
	IR	MD22	*IAAV1101/BS00022966_51*	143.8		4.4	10.0	L
*QLr.spa-4A*	SEV	MD19	*BobWhite_c48455_818*	32.2	536,615,437	4.8	11.0	L
	IR	MD19	*BobWhite_c48455_818*	32.2		6.3	14.1	L
	SEV	MD21	*BobWhite_c48455_818*	32.2		4.3	10.0	L
	IR	MD21	*BobWhite_c48455_818*	32.2		2.7	6.4	L
	SEV	MD22	*BobWhite_c48455_818*	33.2		4.7	10.7	L
	IR	MD22	*RAC875_c27704_420*	32.1		3.2	7.6	L
	IT	12-3 MBDS	*BobWhite_c48455_818*	32.2		102	95.1	L
	IT	74-2 MGBJ	*BobWhite_c48455_818*	32.2		102	95.7	L
	IT	06-1-1 TDBG	*BobWhite_c48455_818*	32.2		66.2	87.4	L
	IT	77-2 TJBJ	*BobWhite_c48455_818*	32.2		99.8	93.9	L
	IT	21-84-1 TCTS	*BobWhite_c48455_818*	32.2		34.1	56.2	L
*QLr.spa-4B*	SEV	MD19	*IAAV2725*	128.9	644,407,948	5.1	11.9	G
	IR	MD19	*IAAV2725*	128.9		5.4	12.5	G
	SEV	MD21	*IAAV2725*	128.9		4.5	10.5	G
	IR	MD21	*IAAV2725*	128.9		3.5	8.3	G
	SEV	MD22	*IAAV2725*	128.9		4.8	11.1	G
*QLr.spa-5A*	SEV	MD19	*BobWhite_c9057_118*	169.7	504,382,440	3.0	7.1	L
	IR	MD19	*BobWhite_c9057_118*	169.7		3.4	7.9	L
	SEV	MD21	*BobWhite_c9057_118*	169.7		5.8	13.2	L
	IR	MD21	*BobWhite_c9057_118*	169.7		6.2	14.0	L
	SEV	MD22	*BobWhite_c9057_118*	*169.7*		3.4	7.8	L
	IR	MD22	*BobWhite_c9057_118*	169.7		4.0	9.2	L
*QLr.spa-7A*	IT	06-1-1 TDBG	*BS00020236_51*	295.7	737,271,531	7.4	16.5	L
	SEV	MD19	*Excalibur_c3476_691*	307.64	744,474,903	3.4	8.0	L
	IR	MD19	*Excalibur_c3476_691*	307.64		3.0	7.1	L
	SEV	MD21	*Excalibur_c3476_691*	307.64		3.8	8.9	L
	IR	MD21	*Excalibur_c3476_691*	307.64		3.4	8.0	L
	SEV	MD22	Excalibur_c3476_691	307.64		1.5	3.6	L
	IR	MD22	*Excalibur_c3476_691*	307.64		1.8	4.3	L
*QLr.spa-7B*	SEV	MD19	*RFL_Contig3005_1031*	12.643	756,750,226	6.9	14.3	G
	IR	MD19	*RFL_Contig3005_1031*	12.643		8.1	16.6	G
	SEV	MD22	*RFL_Contig3005_1031*	12.643		10.1	21.7	G
	IR	MD22	*RFL_Contig3005_1031*	12.463		8	18.1	G

a IT, infection (pustule) type at seedling (isolate listed); IR, infection response in the field; SEV, field disease severity.

b Physical locations of Illumina 90 K SNP markers in the IWGSC RefSeq v2.1 genome assembly (Zhu et al. 2021).

c P, AAC Prevail; B, BW961; H, CDC Hughes; C,AAC Concord; L, Lillian; G, Glenlea.

**Table 4:** Summary of QTL that were identified in the AAC Prevail/BW961, CDC Hughes/AAC Concord, and Lillian/Glenlea populations and their likely correspondence with cataloged *Lr* genes.

	1DS	2BS	2DS	3DL.1	3DL.2	4AL	4BL	5AL	7AL	7B	7DS
BW961			3×^b^								3×
AAC Prevail						3×					
AAC Concord				2×		3×			3×		
CDC Hughes	2×				3×						
Lillian		3×				3×		3×	3×		
Glenlea							2×			2×	
Resistance type^a^	ASR	APR	ASR	ASR	APR	ASR	APR	APR	ASR	APR	APR
Likely gene	*Lr21*	*Lr13*	*Lr2a*	*Unknown*	*Unknown*	*Lr30*	*Unknown*	*Unknown*	*LrCen*	*Unknown*	*Lr34*

^a^ASR, all-stage resistance; APR, adult plant resistance.

^b^The 2× and 3× refer to the number of environments in which each QTL was significant and are located in a cell that indicates the contributing parent and chromosome.

For the HC population, four QTL located on chromosomes 1DS, 3DL.1, 4AL, and 7AL were revealed with the seedling data. The 1DS QTL was detected with races 12-3 MBDS, 128-1 MBRJ, 74-2 MGBJ, 06-1-1 TDBG, and 77-2 TJBJ but was not effective against 21-84-1 TCTS. The QTL explained a phenotypic variation of 11.0% to 38.8%. The QTL on 3DL.1 was derived from AAC Concord and was effective against 12-3 MBDS, 128-1 MBRJ, and 74-2 MGBJ. The 4AL QTL, inherited from AAC Concord, was effective against *Pt* races 12-3 MBDS, 06-1-1 TDBG, 21-21-1 TNBJ, and 77-2 TJBJ, explaining 12.7% to 68.9% of the phenotypic variation. The 7AL QTL associated only with race 06-1-1 TDBG was inherited from AAC Concord and explained 13.3% of the phenotypic variation in the seedling infection.

For the LG population, a seedling QTL was located on 4AL inherited through the Lillian parent effective against *Pt* isolates 12-3 MBDS, 06-1-1 TDBG, 74-2 MGBJ, 77-2 TJBJ, and 21-84-1 TCTS, with the explained variation in the phenotype ranging from 56.2% to 95.7% but not effective against 128-1 MBRJ. This corresponded with a field QTL on 4AL from Lillian in this population. There was also a seedling QTL associated only with 06-1-1 TDBG isolate resistance on 7AL in Lillian that corresponded with the field QTL on 7AL.

### QTL detected using field adult plant response data

The adult plant leaf rust data of the PB population revealed three significant QTL (**Tables 3**, **4**). QTL on chromosome 4AL (designated as *QLr.spa-4A*) with the logarithm of odd (LOD) values 2.1–9.5 and resistance alleles from AAC Prevail, and on chromosome 2DS (*QLr.spa-2D*) with LOD values 2.1–4.7 and on 7DS (*QLr.spa-7D*) with LOD values 5.3–8.9 contributed by BW961 were associated with a significant reduction in leaf rust severity and infection response in the population. The *QLr.sparc-4A* was significant at all three test environments, with an explained phenotypic variation for disease severity and infection response ranging from 5.8% to 17.6%. The *QLr.spa-2D* was significant for disease severity in all environments and infection response in one environment, with the variation explained ranging between 4.2% and 9.1%. A third QTL, *QLr.spa-7D*, was significant in all three tests associated with disease severity and infection response and explained 10.3% to 16.5% of the phenotypic variation.

In the HC population, four QTL were detected with the field leaf rust data (**Tables 3**, **4**). Two of the QTL located on chromosomes 1DS (*QLr.spa-1D*) with LOD values 2.2–8.5 and 3DL.2 (*QLr.spa-3D*) with LOD values 2.5–4.4 were derived from CDC Hughes. The other two QTL on 4A (*QLr.spa-4A*) with LOD values 6.7–10.9 and 7AL (*QLr.spa-7A*) with LOD values 1.2–4.1 were derived from AAC Concord. The *QLr.spa-1D* was significant in two out of three environments, with the explained variation in disease severity and infection response ranging from 5.2% to 18.7%. *QLr.spa-3D* was expressed in all three tests and explained 5.3% to 10.1% of the variation. The *QLr.spa-4A* was significant in two out of three environments associated with disease severity and infection response and explained a phenotypic variation of 15.1% to 23.5% in the disease traits. The *QLr.spa-7A* was significant in all environments and explained 2.8% to 9.5% of the phenotypic variation.

For the LG population, the field phenotypic data revealed six QTL (**Tables 3**, **4**) associated with leaf rust resistance. The resistance alleles for four of the QTL located on chromosomes 2BS (*QLr.spa-2B*), 4AL (*QLr.spa-4A*), 5AL (*QLr.spa-5A*), and 7AL (*QLr.spa-7A*) were inherited from Lillian. The remaining two were derived from Glenlea detected on 4BL (*QLr.spa-4B*) and 7BL (*QLr.spa-7B*). All the QTL derived from Lillian were significant across all environments with a few exceptions, and the explained phenotypic variation ranged between 3.6% and 16.5% across traits. The *QLr.spa-4B* was significant across all the years, and *QLr.spa-7B* was significant in two out of three environments. The explained variation in the leaf rust phenotype for all the Glenlea-derived QTL ranged between 8.3% and 21.7%.

## Discussion

### QTL in the AAC Prevail/BW961 population

The Morden 2021 trials were affected by dry weather, as reflected in the intermediate correlation values of the leaf rust data with other years (**Table 2**). However, this intermediate value of the correlation indicates the consistency in the expression of the leaf rust resistance genes/QTL across variable environments. The susceptibility of AAC Prevail over BW961 in the year 2021, which was characterized by dry weather as opposed to the two wetter years 2019 and 2020, could be attributed to the interactions between the resistance genes and the environment. Despite the detection of only three QTL in the PB population, the skewness of the disease severity distribution with a preponderance of resistant lines exhibited in the populations shows the effectiveness of various combinations of these resistance genes.

AAC Prevail only donated a single QTL for resistance, *QLr.spa-4A*, in the PB population, compared with two from BW961. *QLr.spa-4A* was coincident and mapped to very similar locations in the other crosses with this gene inherited from AAC Prevail, AAC Concord, and Lillian. Since the effect of this gene was consistent in its expression in different genetic backgrounds and multiple environments, *QLr.spa-4A* is valuable in developing new leaf rust-resistant cultivars. Comparing the physical position of markers associated with the QTL in three populations, *Tdurum_contig32577_286* placed at 569.2 Mb, *wsnp_Ex_c2266_4247520* at 522 Mb, and *BobWhite_c48455_818* at 536.6 Mb in the IWGSC RefSeq v2.1 Chinese Spring (CS) reference sequence (Zhu et al., 2021) suggested they are associated with the same QTL region. The identity *QLr.spa-4A* is not known, but *Lr30*, which is found in the Canadian wheat cultivar Pasqua (Dyck, 1993), is located on 4AL. All the cultivars AAC Prevail, AAC Concord, and Lillian are descendants of Pasqua (**Figure 1**), and they most likely inherited *Lr30* from Pasqua. In seedling tests of five Canadian *P. triticina* isolates, all those avirulent to *Lr30* were also avirulent to Lillian. Additionally, when a wider number of diverse Canadian isolates virulent to *Lr30* were tested they were also virulent to Lillian. This gene was also effective at the seedling stage to *Lr30* avirulent isolates but not to the single *Lr30* virulent isolate used in 128-1 MBRJ. The similarity in the effectiveness, chromosomal location, reaction to a wide range of *P. triticina* isolates, and pedigree information suggests that *QLr.spa-4A* and *Lr30* are most likely the same.

The leaf rust resistance of BW961 was attributed to QTL located on 2DS (*QLr.spa-2D*) and *QLr.spa-7D* (*Lr34*). The *QLr.spa-2D* conditioned a nearly immune “0” response to the 12-3 MBDS, 128-1 MBRJ, and 74-2 MGBJ isolates but a 2 or 3 response to the 06-1-1 TDBG and 77-2 TJBJ isolates, typical of *Lr2a*. Comparing the map position of *QLr.spa-2D* with *Lr2a*, the *QLr.spa-2D* closest marker, *RAC875_c65419_229*, was reported for its association with *Lr2a* in the Canadian wheat cultivar Superb (Lewarne, 2021). Also, *Excalibur_c1944_1017*, a marker flanking *QLr.spa-2D*, and *RAC875_c65419_229* flanking both *QLr.spa-2D* and *Lr2a*, are physically positioned at 63.7 Mb in the Wheat Chinese Spring IWGSC RefSeq v2.1 genome assembly (Zhu et al., 2021). *Lr2a* is a common gene in the Canadian wheat germplasm (McCallum et al., 2016a). The wide presence of *Lr2a* in the Canadian wheat cultivars, its chromosomal location, and the results of the *Pt* race analysis suggest the *QLr.spa-2D* in BW961 could be *Lr2a*. *BobWhite_c40479_283*, the marker associated with the *QLr.spa-7D* QTL in BW961 with the current study, was reported by Bokore et al. (2020) for its association with the *Lr34* gene in cultivar Lillian. Based on markers linked with the QTL, pedigree information, field response, and the lack of any associated seedling resistance gene, the BW961-derived adult plant resistance gene *QLr.spa-7D* is the same as *Lr34*.

### QTL in the CDC Hughes/AAC Concord population

Compared with the rest of the cultivars studied here, CDC Hughes had relatively high disease severity rated from 47.5% to 72.5% across the three environments, which contrasts with the moderate resistance reported during its registration. Two QTL, the first at 1DS (*QLr.spa-1D*) conditioning seedling-type resistance and the second at 3DL (*QLr.spa-3D.2*) conditioning adult-type resistance, were responsible for the resistance in CDC Hughes. The *QLr.spa-1D* corresponded with the *Lr21* gene, which is common in Canadian wheat cultivars (McCallum et al., 2016a). In a hexaploid wheat consensus map involving 14 Canadian wheat genetic populations (Bokore et al., 2020), markers associated with the 1DS QTL in CDC Hughes *RAC875_c48669_292* and *BS00093336_51* are within 2.74–3.28 cM map distance from markers *BobWhite_c4303_524* and *RAC875_c2070_566* linked with the *Lr21* gene in the wheat cultivar Vesper (Bokore et al., 2020). *Puccinia triticina* virulence against *Lr21* was observed in 2011 for the first time in Canada (McCallum et al., 2017) and in 2010 in the USA (Kolmer and Anderson, 2011), with the virulence levels fluctuating from year to year (McCallum et al., 2021) and *Lr21* contributing various levels of protection. Likewise, the 1DS gene in CDC Hughes explained 5.1% to 18.7% of the phenotypic variation in the leaf rust field response across the Morden 2020 and 2021 environments with non-significant expression in 2019. The *QLr.spa-1D* also corresponded with seedling resistance to the *Lr21* avirulent isolates 12-3 MBDS, 128-1 MBRJ, 74-2 MGBJ, 06-1-1 TDBG, and 77-2 TJBJ but did not have an effect on the *Lr21* virulent isolate 21-84-1 TCTS. The gene located on 3DL.2 (*QLr.spa-3D.2*) in CDC Hughes conditions adult plant resistance as opposed to the *QLr.spa-3D.1* in the AAC Concord that expressed seedling-type resistance but was not effective on adult plants. *QLr.spa-3D.2* could be a novel gene as there was no *Lr* gene cataloged on the chromosome arm.

AAC Concord contributed QTL *QLr.spa-3D.1*, *QLr.spa-4A*, and *QLr.spa-7A*. The *QLr.spa-4A* was in common with AAC Prevail, AAC Concord, and Lillian and is thought to be *Lr30* as described above. The *QLr.spa-7A* in AAC Concord was also in common with Lillian. This resistance gene is thought to be *LrCen*, a resistance gene commonly found in Canadian wheat cultivars that is only effective against a small number of *P. triticina* races (McCallum and Hiebert, 2012). It has a characteristic mesothetic or *X* infection type. This gene was effective against 06-1-1 TDBG, producing a mesothetic "X" infection type, and a seedling QTL corresponding to this isolate mapped to the same location as the field QTL. Located in a similar genomic region, the *QLr.spa-7AL* in Lillian associated with *Excalibur_c3476_691* placed at 744.5 Mb and *BS00020236_51* at 737 Mb in IWGSC RefSeq v2.1 (Zhu et al., 2021) and in AAC Concord associated with *RAC875_c90330_82* at 727.4 Mb and *BS00068033_51* at 727.6 Mb, indicating the same locus. The *QLr.spa-7AL* in AAC Concord might be inherited from Lillian, as AAC Concord was selected from the cross of Lillian with two other lines (**Figure 1**).

The *QLr.spa-3D.1* was located on the long arm of the chromosome. Among designated *Lr* genes, the seedling gene *Lr24* derived from *Agropyron elongatum* (3DL/3Ag translocation) is similarly located on 3DL and tightly linked with the stem rust resistance gene *Sr24* (McIntosh et al., 1977; Schachermayr et al., 1995; Dedryver et al., 1996). The *QLr.spa-3D.1* QTL source parent, AAC Concord, is not genetically related to *A. elongatum*, indicating the QTL is different from *Lr24* (Crop Information Engine and Research Assistant (CIERA)) (**Figure 1**). *Lr24* and *Lr16* in combination with additional adult plant resistance genes have been reported to give high resistance in Canada and the USA early in the 2000s (Oelke and Kolmer, 2004). In contrast, *QLr.spa-3D.1* expressed seedling resistance but not effective field resistance, indicating its insignificance in resistance breeding.

### QLT in the Lillian/Glenlea population

The Glenlea QTL *QLr.spa-4B* and *QLr.spa-7B* are different from genes previously reported in the cultivar (Dyck et al., 1985; McCallum et al., 2012). The *Lr* genes Dyck et al. (1985) reported in Glenlea *Lr1*, *LrT2=Lr34*, and an allele of, or a gene closely linked to, *Lr13* were not detected in the current study. *Lr1* is ineffective in the field, and all isolates used were virulent on *Lr1*, so it was not detected, though it would have segregated in this population. *Lr34* is carried by both Glenlea (Dyck et al., 1985) and Lillian and is therefore fixed in this population (Randhawa et al., 2013; Bokore et al., 2020). *QLr.spa-4B*, located on the long arm of 4B, is associated with peak marker *IAAV2725* located at 644.4 Mb in the Chinese spring wheat physical map. Two designated genes *Lr12* and *Lr49* have been reported on the 4B long chromosome arm. Another Canadian wheat cultivar, AAC Domain, has been hypothesized to have the adult plant gene *Lr12* (Liu and Kolmer, 1997); however, virulent *Pt* races exist for this gene in Canada (McCallum et al., 2021). The adult plant resistance gene *Lr49* could correspond with *QLr.spa-4B* based on the relative position of markers associated with genes on both the physical map and consensus map (Bokore et al., 2020). The *Lr49* flanking markers *Xbarc163* located at 607.1 Mb and *Xwmc349* at 639.9 Mb (Bansal et al., 2008) and Excalibur_c47209_87 at 603.1 Mb and IACX938 at 610.3 Mb (Nsabiyera et al., 2019) are 4.5–37.3 Mb from the *QLr.spa-4B* marker *IAAV2725* in the physical map and 6.3–23.2 cM from *IAAV2725* on the consensus map (Bokore et al., 2020). The presence of *Lr49* in Canadian wheat germplasm is unknown, but the close proximity of markers flanking the gene and *QLr.spa-4B* suggests both represent the same gene or two genes located in a similar genomic region.

Comparing the genetic map of different mapping populations and the physical positions of the QTL-associated SNP markers, the Glenlea-derived 7BL QTL is located in the same genomic region with a QTL, *QLr.spa-7B.2*, identified in three Canadian cultivars, namely AC Cadillac, Vesper, and Red Fife (Bokore et al., 2020). Additionally, markers associated with the Glenlea 7BL QTL and *QLr.spa-7B.2* in AC Cadillac, Vesper, and Red Fife are closely located in the hexaploid wheat consensus map (Wang et al., 2014). Resistance genes on 7BL (Mcintosh et al., 2014) include *Lr14a* (seedling gene) and *Lr68* (adult plant gene), but these were not detected in Glenlea previously. Given that the LG cross is fixed for the presence of *Lr34*, additional QTL in this cross may be detected because of their interaction with *Lr34*, which is known to enhance the effects of other resistance genes. The seedling resistance phenotype of the 4AL (*Lr30*) QTL was more resistant in this cross than in the other two crosses, likely because *Lr34* enhanced the effect of this resistance gene at the seedling stage.

The cultivar Lillian has remained effectively resistant against leaf rust since the time of its release owing to the resistance conditioned by genes located on 2BS, 5AL, and 7AL identified in the current study, 4AL identified in this and a previous study (Bokore et al., 2020), and *Lr34* (Randhawa et al., 2013; Bokore et al., 2020). The 2BS QTL cannot be *Lr16*, similarly located on the short chromosome arm, because the *Pt* races used for the seedling test were avirulent on *Lr16*, but all the lines without the 4AL gene were found to be susceptible to most races, and no seedling QTL was found at this location. In contrast, the 2BS QTL could correspond with the adult plant gene *Lr13*, which is common in Canadian wheat germplasm. For example, markers linked with *Lr13* in Carberry *Excalibur_c45094_602* and *Excalibur_rep_c106124_239* are located at 103.5 Mb physical position like the Lillian 2BS markers *IAAV1101 and BS00022966_51.*

The Lillian adult plant QTL on 5AL (*QLr.spa-5A*) likely is the same as the QTL inherited by Carberry in Carberry/AC Cadillac and Carberry/Thatcher populations (Bokore et al., 2020; Bokore et al., 2022). *QLr.spa-5A*, in Lillian and Carberry, was associated with markers that are located adjacent to each other in the IWGSC V2.1 (Zhu et al., 2021). Accordingly, the Carberry *QLr.spa-5A*-associated marker *Kukri_rep_c104877_2166* positions at 480.8 Mb (Bokore et al., 2022) and *BobWhite_c1387_798* at 528.8 Mb (Bokore et al., 2020), and the Lillian *QLr.spa-5A* marker *wsnp_Ex_c39592_46849607* positions at 477.1 Mb, *BS00010698_51* at *537.5* Mb, and *wsnp_Ku_c12211_19780409* at 550.0 Mb. Also, these markers shared the same 5AL chromosomal region (55–75 cM) in the hexaploid consensus map (Wang et al., 2014). The *QLr.spa-5A* gene was not segregating in the crosses of Vesper with Lillian or Carberry (Bokore et al., 2020), suggesting Vesper similarly has the same gene as Lillian and Carberry, indicating the wide presence of the gene in Canadian wheat breeding lines. Also, the *QLr.spa-5A* gene/QTL could be considered new as there was no designated gene reported on chromosome arm 5AL (Mcintosh et al., 2014; Kumar et al., 2022).

Out of six cultivars studied here, Glenlea and Lillian are the only cultivars that have been analyzed for their leaf rust resistance previously (Dyck et al., 1985; Bokore et al., 2020). Interestingly, Glenlea is an old wheat line registered in 1972 (Evans et al., 1972), but it maintained an intermediate level of resistance against leaf rust for a long time. The Glenlea resistance is a combined effect of genes identified in the current study in the cultivar, *QLr.spa-4B* and *QLr.spa-7B*, and previously reported genes *Lr34*, *Lr1*, and *LrT2* (Dyck et al., 1985, McCallum et al., 2012). Lillian is resistant to the leaf rust that has been maintained since its release in 2003 (DePauw et al., 2005), but only four significant genes could be identified from the cultivar, with the QTL on 2BS (likely *Lr13*), 4AL (*Lr30*) and 5AL segregating in the LG population, and the 4AL (*Lr30*) and 7DS (*Lr34*) in the Vesper/Lillian population (Bokore et al., 2020).

Besides Glenlea and Lillian, we identified several resistance genes in AAC Concord on 3DL, 4AL (*Lr30*), and 7AL (*LrCen*), AAC Prevail on 4AL (*Lr30*), CDC Hughes on 1DS (*Lr21*) and 3DL, and BW961 on 2DS (*Lr2a*) and 7DS (*Lr34*). With the exception of the 4AL QTL from Lillian, AAC Prevail, and AAC Concord and the 7AL from Lillian and AAC Concord, the QTL identified in the current study were confined to single cultivars. In conclusion, the results of this study will widen our knowledge of leaf rust resistance genes in Canadian wheat and their utilization in resistance breeding. Kompetitive allele-specific PCR (KASP) markers linked with the genes identified have been developed for use in marker-assisted breeding.

## Data availability statement

The original contributions presented in the study are included in the article/**Supplementary Material**. Further inquiries can be directed to the corresponding authors.

## Author contributions

RC and RK developed the mapping populations. RC, RK, BDM, CH, and FB conceived, designed, and supervised the execution of the study. BDM, FB, RK, RC, and SB performed the field trials and leaf rust phenotyping. BDM conducted seedling resistance trials. FB, BM, and CH performed genetic data mining and linkage genetic mapping. FB identified SNP primers for KASP marker designing. CH developed and validated KASP markers. FB performed QTL analysis, summarized and interpreted the results, and wrote the draft paper. BDM revised the draft. All the authors reviewed the paper. All authors contributed to the article and approved the submitted version.

## Supplementary material

Supplementary Table 1 Number of lines (N), mean, range, and standard deviation (STDEV.) of leaf rust severity and infection responses for three doubled haploid wheat populations evaluated at Morden, MB in different years. ^a^LRS = leaf rust severity in percent; LRIR = leaf rust infection response. ^b^CV = coefficient of variation.

Supplementary Table 2 Genotype data of the three mapping populations (excel sheet attached).
