# Supplementary material for: Genetic mapping of leaf rust (Puccinia triticina Eriks) resistance genes in six Canadian spring wheat cultivars
Source: Front Plant Sci. 2023 Mar 20;14:1130768. doi: 10.3389/fpls.2023.1130768 (PMC10067638; doi:10.3389/fpls.2023.1130768)
Supplement: Supplementary Table 1 — Number of lines (N), mean, range, and standard deviation (STDEV.) of leaf rust severity and infection responses for three doubled haploid wheat populations evaluated at Morden, MB in different years. aLRS = leaf rust severity in percent; LRIR = leaf rust infection response. bCV = coefficient of variation. [file Table_1.docx]

**Supplemental** **Table S1.** Mean, range and standard deviation (STDEV) of leaf rust severity and infection response of three doubled haploid wheat populations evaluated at Morden, MB in different years.

|  | **Population mean and range** | | | | **DH parent** | | **Susceptible check** |
| --- | --- | --- | --- | --- | --- | --- | --- |
| **AAC Prevail/BW961 (PB)** | **Mean** | **STDEV** | **CV** ^b^ | **Range** | **AAC Prevail** | **BW961** | **Thatcher** |
| LRS MD 2019 ^a^ | 20.4 | 18.6 | 0.9 | 0.5 – 80.0 | 13.3 | 25.8 | 77.5 |
| LRS MD 2020 | 14.4 | 17.7 | 1.2 | 0.0 - 67.5 | 7.5 | 13.1 | 82.5 |
| LRS MD 2021 | 31.4 | 20.9 | 0.7 | 1.0 – 85.0 | 46.9 | 28.1 | 76.7 |
| LRIR MD 2019 |  |  |  | R - MSS | MR | MS | S |
| LRIR MD 2020 |  |  |  | R - MSS | MR | MRMS | S |
| LRIR MD 2021 |  |  |  | R - MSS | MRMS | MRMS | MS |
| **CDC Hughes/AAC Concord (HC)** | **Mean** | **STDEV** | **CV** | **Range** | **AAC Concord** | **CDC Hughes** | **Thatcher** |
| LRS MD 2019 | 21.2 | 20.9 | 1.0 | 0 .0 - 90.0 | 5.0 | 72.5 | 87.5 |
| LRS MD 2020 | 9.4 | 16.3 | 1.7 | 0.0 – 65.0 | 0.8 | 52.5 | 85.0 |
| LRS MD 2021 | 24.9 | 18.6 | 0.7 | 1.0 – 80.0 | 13.8 | 47.5 | 60.0 |
| LRIR MD 2019 |  |  |  | R - S | MR | S | S |
| LRIR MD 2020 |  |  |  | R - S | MR | MRMS | S |
| LRIR MD 2021 |  |  |  | R - S | RMR | MS | MS |
| **Lillian/Glenlea (LG)** | **Mean** | **STDEV** | **CV** | **Range** | **Glenlea** | **Lillian** | **Thatcher** |
| LRS MD 2019 | 20.8 | 19.3 | 0.9 | 0.0 – 80.0 | 35.0 | 7.5 | 90.0 |
| LRS MD 2021 | 11.8 | 10.1 | 0.9 | 0.0 – 40.0 | 31.3 | 2.0 | 58.8 |
| LRS MD 2022 | 25.9 | 21.0 | 0.8 | 0.0-80.0 | 38.8 | 13.8 | 80.0 |
| LRIR MD 2019 |  |  |  | R - MSS | MS | MR | MSS |
| LRIR MD 2021 |  |  |  | R - MRMS | MRMS | R | MS |
| LRS MD 2022 |  |  |  | R - S | MRMS - S | MR - MRMS | MSS - S |

^a^ LRS = leaf rust severity in percent; LRIR = leaf rust infection response.

^b^ CV = coefficient of variation.
